# A Meta-Analysis to Understand the Relationship between Pig Body Weight and Variation from Birth to Market

**DOI:** 10.3390/ani11072088

**Published:** 2021-07-14

**Authors:** Andres F. Tolosa, Joel M. DeRouchey, Mike D. Tokach, Robert D. Goodband, Jason C. Woodworth, Jordan T. Gebhardt, Mathew J. Ritter, Chad M. Pilcher

**Affiliations:** 1 Department of Animal Sciences and Industry, Kansas State University, Manhattan, KS 66506, USA; jderouch@ksu.edu (J.M.D.); mtokach@ksu.edu (M.D.T.); goodband@ksu.edu (R.D.G.); jwoodworth@ksu.edu (J.C.W.); 2 Department of Diagnostic Medicine/Pathobiology, Kansas State University, Manhattan, KS 66506, USA; jgebhardt@vet.k-state.edu; 3 Provimi North America, Lewisburg, OH 45338, USA; Mritter@provimi-na.com (M.J.R.); cpilcher@provimi-na.com (C.M.P.)

**Keywords:** pigs, meta-analysis, coefficient of variation, standard deviation

## Abstract

**Simple Summary:**

Understanding and managing variation in live weight within a pig population is key for swine producers to avoid economic penalties at processing plants. The objective of this meta-analysis was to determine the relationship between coefficient of variation (CV) and standard variation (SD) as a function of body weight for pigs and develop equations to predict CV and SD of a population of pigs from birth to market weight. Results reveal that there is a quadratic relationship between variation and body weight. Coefficient of variation decreases as live weight increases, but the slope is less pronounced as body weights became greater. Conversely, SD increases quadratically as body weight (BW) increases, with a less pronounced slope when BW is high within the population. Thus, the equations developed can be an effective tool for producers to predict normal BW variation within a group of pigs, which can then aid in the development of marketing strategies for finishing pigs.

**Abstract:**

This meta-analysis aims to understand the changes in pig body weight (BW) variation from birth to market and develop prediction equations for coefficient of variation (CV) and standard deviation (SD) as a function of BW. Standard deviation is the measure of dispersion of a set of values from the mean and CV is the SD expressed as a percentage of the mean. Data collected from 16 papers and data sets yielded 117,268 individually weighed pigs with sample size ranging from 120 to 4108 pigs. Polynomial regression analysis was conducted separately for each variation measurement. The resulting prediction equations (CV (%) = 20.04 − 0.135 × (BW) + 0.00043 × (BW)^2^, R^2^ = 0.79; SD = 0.41 + 0.150 × (BW) − 0.00041 × (BW)^2^, R^2^ = 0.95) suggest that there is a quadratic decreasing relationship between the CV of a population and BW, the slope gets smaller as mean BW increases from birth to market. A quadratic increasing relationship is observed for SD, with slope being smaller as mean BW of pigs increases from birth to market. These prediction equations can be used by swine producers to estimate expected CV and SD of BW among a population of pigs.

## 1. Introduction

The swine industry has been constantly evolving to raise pigs with improved growth performance. However, pig body weight variation remains a problem for producers. Packing plants require that pigs are marketed within certain weight allowances and when pigs fall outside the packer’s desired weight range, they are discounted in market value, resulting in negative economic consequences for swine production systems. Therefore, it has been of continual interest for producers and researchers to understand how pig body weight variation changes from birth to market weight.

Previous research regarding variation from birth to market is unclear and somewhat inconsistent in the relationship between variation in birth weight and variation in post-natal growth performance [1]. When there is high variation in body weight within a population, lighter pigs can have a disadvantage. When placing light pigs with heavy pigs, competition over milk from the sow or feed in the post-weaning period can result in unequal growth among contemporaries. It has been observed that light birth weight pigs are less successful in competing for milk when compared with heavier littermates [2]. Additionally, previous research has reported a strong relationship between weaning weight and subsequent growth performance, which can be an influencing factor on BW variation in early stages of growth [3,4,5,6]. Wellock [6] reported that after weaning, variation in the growth response is also affected by stressors when pigs were housed in commercial environments. Therefore, unequal growth rate within a group of pigs post-weaning can lead to increased body weight variability. As a result, efficiency of facility utilization throughout the production cycle is decreased [7].

Data are well established that sorting pigs into uniform weight pens does not improve overall population performance [1,8,9,10]. Camara [9] concluded that regrouping pigs at weaning to uniform BW groups had no influence on growth performance. Similarly, for the grow–finish period, sorting pigs into lower within-pen body weight (BW) variation has been studied. O’Quinn [10] reported that sorting uniformly by BW (i.e., reduced within-pen weight variation) has little effect on improving overall performance or variability in individual final body weight. Therefore, understanding pig growth and body weight variation is essential to lessen the impacts of increased variation in final live weight.

The aim of this systematic review and meta-analysis was to evaluate body weight variation focused on the mean of an entire population of pigs, and to develop regression equations to predict pig body weight variation, such as coefficient of variation and standard deviation as a function of body weight.

## 2. Materials and Methods

### 2.1. Literature Search

A literature search was conducted via Cab Direct using Kansas State University Libraries using the keywords “variation of pigs”, “coefficient of variation and pigs”, “standard deviation and pigs”, or “variation in pigs from birth to market”. Data were derived from refereed and nonrefereed publications, including theses, technical memos, and university publications. The published literature that was used reported measurements of variability where individual pig starting and ending weights were collected from birth to market. Interim weight periods were also used, if available.

### 2.2. Selection for Inclusion and Exclusion Criteria

For inclusion, publications were reviewed to determine if the pig populations had been artificially selected to reduce variation (i.e., light pigs separated from heavy pigs). When the procedures described segregation or selection of the pigs within the population with the goal of forming uniform groups per treatment, data were excluded. Additionally, trials were not included if management strategies were applied to the population or part of the population in order to decrease BW variability within the group of pigs. Other information included during the data extraction process were trial sample size and initial and final BW. Finally, Cook’s distance diagnosis analysis was used to identify data points that negatively affect the regression model. A large number indicates that the observed value has strong influence on the estimated coefficients for the regression analysis. Cook’s distance is considered the single most representative measure of influence on overall fit. It captures the impact of an observation from two sources: (1) size of changes in the predicted values when the value is omitted and (2) the observation’s distance from the other observations (leverage). These influential data points could be considered outliers (data points with large residuals) [11]. From the initial 25 trials, 9 trials were excluded due these criteria. Thus, 16 trials published in journal articles, technical memos, and theses were used (Table 1) for final analyses.

### 2.3. Data Base Preparation and Meta-Analysis

Data from each trial were recorded in a spreadsheet template. Data included study name, sample size, body weight (BW), and measurements of variation. For papers that reported coefficient of variation, standard deviation was calculated and vice versa. The final database yielded a total of 204 observations, including 117,268 individually weighed pigs. Sample size within each data set ranged from 120 to 4108 pigs. Pigs used in the database had BW ranging from 1.36 to 175 kg.

A meta-analysis in R [12] was carried out using a random effects model to account for both within- and between-study variances. The effect size (i.e., the summary proportion) was estimated through the DerSimonian and Laird method [13] with the assumption that the different studies were estimating different, yet related, effects on variation. Trial was included as a random effect and a weight parameter was used to account for the sample size of each study. This weighting parameter was the inverse of the standard error of the mean squared to allow larger studies to have more weight and greater impact on the overall mean.

**Table 1:** Summary of publications used in the meta-analysis to predict measurements of variation from birth to market ^1^.

Publication	Trial	Sample Size	Group ^2^	Average initial BW, kg	Average Final BW, kg	Average CV Start	Average CV End	Average SD Start	Average SD End	Reference
Peterson, 2004 ^3^	1	3132	3	34.0	114.6	12.8	8.2	4.33	9.43	[14]
Peterson, 2004 ^3^	2	3492	3	5.38	107.8	16.8	9.1	0.91	9.81	[14]
Main, 2004	1	2272	4	4.6	110.6	18.4	10.6	0.83	11.61	[15]
Main, 2004	2	3456	3	4.8	115.6	19.8	9.6	0.95	11.24	[15]
Fix, 2010	1	4108	1	4.92	103.12	23.78	13.18	1.17	13.60	[16]
Fix, 2010	2	1052	1	1.54	115.2	23.77	7.71	0.37	8.88	[16]
Beaulieu, 2010	1	1114	3	1.40	97.5	19.9	9.7	0.27	9.34	[17]
Shull, 2013 ^3^	1	120	2	5.7	167.5	20.5	8.7	1.17	14.49	[18]
Shull, 2013 ^3^	2	2240	7	5.8	175.8	19.8	9.4	1.15	15.82	[18]
Shull, 2013 ^3^	3	918	2	1.44	135.3	21.2	10.1	0.30	13.55	[18]
Flohr, 2015 ^3^	1	1092	4	36.3	138.4	14.9	7.2	5.41	9.96	[19]
Zotti, 2017	1	1178	5	1.36	20.7	20.4	16.12	0.28	3.34	[20]
Hastad, 2019 ^3^	1	1032	2	30.7	118.3	15.6	12.5	4.80	14.78	[21]
Hastad, 2019 ^3^	2	1176	2	35.2	118.7	15.9	9.7	5.58	11.53	[21]
Faccin, 2020	1	1176	4	6.1	21.5	20.2	19.1	1.25	4.10	[22]
Williams, 2020	1	1448	5	6.1	124.7	17.8	10.1	1.07	12.57	[23]

^1^ For trials that reported variation by period, each period was identified in the database as a trial. ^2^ Number of groups within trial where measurements of variation were reported as a function of body weight. ^3^ Represents groups sorted by sex; all other groups were mixed sex.

### 2.4. Statistical Analysis

The lm procedure of R was used to develop the polynomial regression equations. The CV or SD of each experiment was the dependent variable for modeling the equation. An exploratory data analysis using a scatter plot was initially used to determine whether there was a relationship between measurements of variation and BW as the variables of interest. Linear and quadratic terms of CV and SD, as a function of average BW, were the variables in the regression analysis. Model significance was assessed through analysis of variance using an F-test, where the significance of the coefficients were assessed through a T-test and was determined significant at *p* < 0.05 for each response variable. Coefficient of determination (R^2^) was used describe the amount of variance in the outcome that was explained by the predictor variables. Model assumptions were checked for normality and constant variance. The studentized residuals plots indicated that normality assumptions were met and no evidence for remaining outliers or heteroscedasticity were observed.

## 3. Results

A summary of the studies used for this meta-analysis is presented in Table 1 and Table S1. The values describe average initial and final BW and measurements of variation (coefficient of variation and standard variation). The studies in the final database comprised a mean BW range of 1.36 to 175.8 kg, where coefficient of variation and standard deviation as a function of BW ranged from 23.8 to 7.2% and from 15.82 to 0.27 kg, respectively. The inspection of residuals vs. predicted values relative to the line of equality suggest the predictions were precise, not biased, and that the model assumptions were reasonably met.

### Regression Analysis

**Figure 1:**
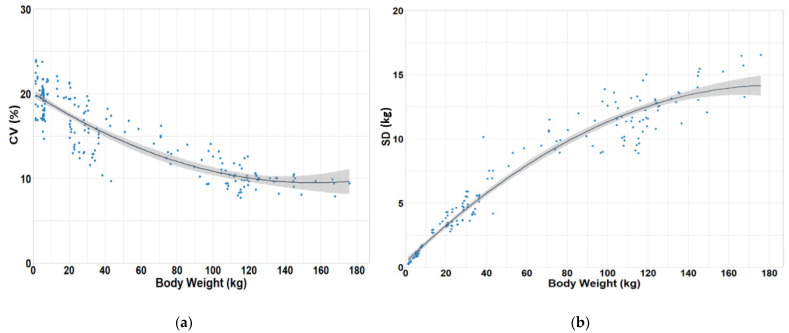
Plots of coefficient of variation (CV) and standard deviation (SD) as a function of body weight (BW) from birth to market. Gray area represents the 95% confidence interval on CV and SD of BW, respectively. (**a**) Relationship between CV and BW (CV = 20.04 − 0.135 BW + 0.00043 BW^2^; standard error of regression coefficients, 0.253, 0.0112, 0.000078, respectively; R^2^ = 0.79, all terms *p* < 0.05). (**b**) Relationship between SD and BW (SD = 0.41 + 0.150 BW − 0.00041 BW^2^; standard error of regression coefficients, 0.133, 0.0059, 0.000041, respectively; R^2^ = 0.95, all terms *p* < 0.05).

The model predicted a quadratic decrease in CV with the most rapid decrease occurring when mean BW is low within the population, with a less pronounced decrease as mean BW increases up to market weight (Table 2 and Figure 1a). Conversely, SD showed a quadratic increase when the population is light and mean BW is low. Subsequently, the slope declines (Table 2 and Figure 1b). The CV model described 79% of the variation in CV, which is explained by the combined linear and quadratic effects of mean group body weight. Similarly, 95% of the variation in standard deviation is explained by the combined linear and quadratic effects of mean group body weight.

## 4. Discussion

There are many factors that affect variation within a population of pigs (e.g., genetic potential, health status of the herd, etc.). In practical situations, when pigs are born, there is a wide distribution of BW that consequently can influence growth rate, but other aspects, such as muscle fiber number, can also affect the development and performance of the pig throughout the production cycle [24,25]. Wolter [25] conducted a literature review and reported that pigs with differences in birth weight of 0.2, 0.5, and 0.7 kg presented differences in post-weaning growth rate of 5, 6, and 8%, respectively. Additionally, the difference in post-natal growth may be a consequence of suboptimal management practices that may result in the pigs born with a lighter BW growing slower when compared with its contemporaries, regardless of an appropriate environment [26].

This meta-analysis showed that variation is high at birth and the distribution of BW within and across litters is wide. However, as the pigs grow from weaning to market, the percent of the population that is furthest from the mean BW of the population starts to decrease. Zotti [20] evaluated potential factors that influence birth weight and within litter heterogeneity and observed a linear effect of sow parity on CV of BW. Sows of parity 5 were observed having 5.4, 6.4, and 4.8% greater CV of BW on day 0, 7, and 21, respectively, when compared with parity 1 sows. However, at the end of the nursery period (day 59), the effect of parity on CV of BW disappeared. Similarly, Beaulieu [17] investigated the effect of litter size on growth performance and CV of BW by evaluating litters in three categories according to the number of pigs per litter, i.e., 3 to 10, 11 to 13, and 14 to 19 pigs per litter, and observed a decrease in birth weight of approximately 33 g per each additional pig born with increasing litter size. Additionally, small litters were observed with a 7.1% higher CV of BW when compared with large litters at birth. However, the greater pre-weaning variation observed in small litters (3 to 10 pigs per litter) when compared with large litters (14 to 19 pigs per litter) was not maintained after weaning. Thus, it is possible that greater variation at birth does not greatly influence the variation of the subsequent periods of growth.

For pigs grouped by weaning weight, a greater variation in growth rate for groups with lower weaning weight than groups with heavier weaning weights can be expected [27]. Thus, a numerical increase in days to market of light pigs when compared with heavy contemporaries can potentially disrupt a continuous flow as pigs are moved through the production system.

Increasing weaning age affects subsequent post-weaning performance. Several studies have evaluated the impact of weaning age on final BW variation. Main [15] reported a decrease in CV of BW at weaning when weaning age increased from 12 to 21 day of age. This reduction in variation was maintained all the way to market, where a decrease of 3.4% in CV of BW was observed for pigs weaned at 21 day when compared with pigs weaned at 12 day of age. Faccin [22] reported a linear increase in average daily gain (ADG) and average daily feed intake (ADFI) that resulted in an improved G:F when weaning age was increased from 19 to 28 day. This increase in performance was maintained up to 136 day after weaning, where BW was observed to increase linearly. Interestingly, CV of BW was affected by weaning age, but this difference between treatments disappeared by day 42 after weaning. Their results may be explained by the linear decrease in removal rate (8.01 to 1.65%) as weaning age increased. Removing light-weight pigs from a group could have resulted in less variation on final BW within the younger weaning age populations. Conversely, Main [15] observed a linear decrease in BW CV on day 42 post-weaning as pigs weaning age increased from 15.5 to 21.5 day. However, this difference in variation at the end of the nursery period did not translate to significant difference in variation at market weight. This also could be in part attributed to a tendency of pigs weaned at 15.5 day to have increased removal rates when compared with pigs weaned on 21.5 day. A key point to understand is that a decrease in BW at weaning across a population can increase the number of light-weight pigs in the population, and, therefore, result in the overall mean BW of a normal distribution curve to shift to the lighter portion of the curve, but the shape of the curve will remain the same. It was observed in our analysis that lighter mean BW represented greater variation (Figure 1a). However, when the population mean BW was increased, the rate of reduction in CV was less pronounced when compared with population of pigs at low BW. Therefore, strategies to increase growth rate of the lighter portion of the population of pigs needs to be prioritized.

Strategies centered on the lighter portion of the population have been evaluated to achieve improvements in overall BW variation. The addition of dietary fat has been observed to improve the gain of light-weight pigs. Hastad [21] sorted pigs by light, mixed, and heavy BW categories at the beginning of the growing–finishing period (approximately 30 to 120 kg) and fed pigs a diet with or without added fat (i.e., 0 or 6%). While heavy pigs had greater ADG and ADFI when compared with the light pigs, the average combined growth performance of these groups was similar to that of mixed BW pigs. The inclusion of dietary fat fed to light-weight pigs improved ADG, concluding that differences in final BW and CV of BW of light pigs when compared with heavy pigs can be reduced when fat is selectively included in the diet fed to the lighter portion of the population.

Diets are commonly formulated to targeted BW ranges and provided as a feed budget with a specific amount of feed required for pigs to achieve the desired weight gain for that BW range. If a group of pigs grow slower, adjustments can be made to assure that pigs are consuming a diet formulated to their specific nutrient requirement before switching to the next diet. Lopez-Verge [11] fed light-weight pigs on a budget or on a fixed time-basis and observed that the pigs fed on a budget were heavier throughout the entire growing–finishing period (18 to 90 kg BW) with a 1.6% reduction on variation (11.3% vs. 9.7% CV of BW) when compared to pigs fed on a fixed time-basis with less days needed to achieve target market BW. In addition, the same study observed that provision of more feeder space (2.2 vs. 5.5 pigs per space) increased ADG, final BW, and numerically decreased CV of BW on 1.7 percent units when compared with pigs raised with less feeder space.

In addition, health issues can have a negative impact in BW variation. Cornelison [28] evaluated two populations that were positive for porcine reproductive and respiratory syndrome virus (PRRSV), a high health challenged population, which was also positive to influenza type A virus, had 5.5% higher CV of BW at weaning when compared to a population with a low health challenge (only impacted by PRRS). This increased variation was maintained until market weight, with the population that was only positive for PRRSV having 3.3% lower CV of BW when compared to the population with a high health challenge (12.2% vs. 15.5% CV of BW).

Thus, pig BW variation within a population can be influenced by health, management, and nutrition. In general, lighter or slower growing pigs need to be prioritized in order to reduce the population’s BW variation. The most practical strategies may need to be combined to have desirable outcomes on BW variation within a pig population. The prediction regression equations that resulted from this analysis are for populations of pigs with a narrow age range. The variation estimates of a population may increase if the age range increases because of extended fill time or due to multiple groups of pigs being placed together over time.

This meta-analysis aims to help understand variation as a function of body weight within a population of pigs. For producers, using these equations to estimate the percent of deviation of the mean body weight together with the kilograms of body weight deviated from overall mean can be beneficial in marketing programs.

Several strategies reviewed in this document show inconsistent responses to influence body weight variation, thus they need to be evaluated closely before implementation. The most practical strategies may need to be combined to have desired outcomes, but research has generally focused on single practices and not combining them to evaluate outcomes. In general, lighter/slow-growing pigs need the most focus to reduce pig population weight variation; however, investment in this subpopulation must be carefully evaluated to have a return an economic investment.

Results of the present study suggest that further research identifying factors that influence variation in pig weight and performance measures and strategies to reduce variation is needed. Experiments reporting different methods to reduce pig BW variation are scarce and their findings are somewhat inconsistent. To the best of our knowledge, this is the first analysis that has summarized the relationships between body weight and measures of variation (CV and SD) as a function of live weight, whereas multiple studies and different populations have been evaluated to achieve a single meaningful prediction equation. Considering packer BW preferences, reducing BW variation allows for pigs to be marketed over a shorter window of time when compared with groups with wide variation. More uniform groups can be more profitable as fewer pigs are sold outside the packer’s BW allowances. Thus, modeling changes in CV and SD as a function of BW will help pork producers with marketing strategies to optimize facility use.

## 5. Conclusions

Results of this meta-analysis indicate that the mean CV of a population had a decreasing quadratic relationship with pig BW. Coefficient of variation decreased as live weight increased, but the slope is less pronounced as mean live weight of the population became greater. Conversely, SD increased quadratically as BW increased, with the slope being less pronounced as BW increased within the population. These prediction equations can be used by swine producers to estimate BW variability more accurately in a population of pigs. Further research is needed to investigate approaches that can assist producers in reducing and managing pig BW variation within a population in order to use facilities more efficiently and market more pigs within a packer-specified ideal BW range.

## Figures and Tables

**Table 2:** Regression equations to predict variation of pigs from birth to market.

Dependent Variable	Independent Variable	Intercept	Live Weight	Live Weight Squared	R Squared ^1^
Coefficient of variation	Live weight, kg	20.04	−0.135	0.00043	0.79
Standard deviation	Live weight, kg	0.41	0.150	−0.00041	0.95

^1^ Coefficient of determination (R^2^).

## Data Availability

Data from this study are available by email request to the corresponding author.

## Supplementary Materials

The following are available online at https://www.mdpi.com/2076-2615/11/7/2088/s1, Table S1: Pig genotype and type of system for trials used in the meta-analysis.
