# Supplementary material for: A Meta-Analysis to Understand the Relationship between Pig Body Weight and Variation from Birth to Market
Source: Animals (Basel). 2021 Jul 14;11(7):2088. doi: 10.3390/ani11072088 (PMC8300151; doi:10.3390/ani11072088)
Supplement: Supplementary file 1 [file animals-11-02088-s001.zip › animals-1225280-SI.pdf]

Suplementary data. Table S1. Pig genotype and type of system for trials used in the meta-analysis.

Publication	Trial	Genotype	Type of system	Reference
Peterson, 2004	1	Mating line A sires, line B sires and line C sires to PIC C-22 dams	Wean-to-finish	[14]
Peterson, 2004	2	Mating line A sires, line B sires and line C sires to PIC C-22 dams	Wean-to-finish	[14]
Main, 2004	1	PIC Line 280 × C22	Multisite production system	[15]
Main, 2004	2	PIC Line 280 × C22	Multisite production system	[15]
Fix, 2010	1	Large White x Landrace sows bred to Duroc boars	Multisite production system	[16]
Fix, 2010	2	Large White x Landrace sows bred to Duroc boars	Multisite production system	[16]
Beaulieu, 2010	1	Camborough Plus, C-22, or F2 sows; by PIC 337 sire	Multisite production system	[17]
Shull, 2013	1	PIC 359 sires mated to PIC C22 or PIC C29 dams	Wean-to-finish	[18]
Shull, 2013	2	PIC 359 sires mated to either PIC C22 or PIC C29 dams	Wean-to-finish	[18]
Shull, 2013	3	PIC 359 sires mated to PIC C29 dams	Farrowing house and then Wean-to-finish	[18]
Flohr, 2015	1	PIC 359 × Genetiporc F25	Wean-to-finish	[19]
Zotti, 2017	1	Topigs Tybor × Topigs 20	Farrowing house and then multisite production system	[20]
Hastad, 2019	1	PIC L337 × 1050	No specified	[21]
Hastad, 2019	2	PIC L337 × 1050	No specified	[21]
Faccin, 2020	1	PIC 337 × Camborough	Multisite production system	[22]
Williams, 2020	1	DNA 241 × 600	Multisite university research facility	[23]
